# Prevalence and Pattern of Ocular Morbidity among School Children in Southern Ethiopia

**DOI:** 10.4314/ejhs.v31i4.18

**Published:** 2021-07

**Authors:** Getahun Abayo, Girum W Gessesse, Tsedeke Asaminew

**Affiliations:** 1 Durame Hospital, Southern Nations, Nationalities and Peoples State, Ethiopia; 2 Department of Ophthalmology, St Paul's Hospital Millennium Medical College, Addis Ababa, Ethiopia

**Keywords:** Ocular morbidities, Visual impairment, blindness, Eye school screening, Dega Kedida, Southern Ethiopia

## Abstract

**Background:**

Visual health is linked to school achievement, quality of life and productivity. But eye screening in school children is not routinely done in most Ethiopian schools. This study was done to determine prevalence and pattern of ocular morbidity among school children in Roman Dega-Kedida, Southern Ethiopia.

**Method:**

This was a cross-sectional study. All children aged 16 years or less, attending the school during the study period were included. Data entry and statistical analyses were performed using SPSS Version 16. A P - value of < 0.05 was considered statistically significant.

**Result:**

A total of 778 children participated in this study. The female to male ratio was 1.06:1. The mean age was 11.34(±2.31) years. A total of 201 (25.8%) children had ocular morbidities. The most common was trachoma found in 85(10.9%). Of these, 49(56.5%) had active trachoma, while 37(43.5%) had either trachoma scar or trachomatous trichiasis. Allergic conjunctivitis was found in 49(6.3%), refractive error in 37(4.8%), and xerophthalmia in 17(2.2%) children. Visual impairment in one or both eyes was found in 41(5.3%) children. Thirty-two children (4.1%) had bilateral Visual impairment of which 6(18.8%) had moderate to severe visual impairment. The cause of visual impairment was refractive error in 37(90.2%), corneal opacity in 3(7.3%) and cataract in 1(2.4%) child.

**Conclusion:**

Most of the ocular diseases observed were either preventable or treatable. Health education to the community and establishing regular school screening program is recommended.

## Introduction

Childhood blindness is one of the priority areas of “Vision 2020: the right to sight”, a global initiative launched by WHO to eliminate avoidable blindness. It has been estimated that 500,000 children become blind every year and about half of them die within one or two years of becoming blind (1). Approximately three-quarters of the world's blind children live in the poorest regions of Africa and Asia. While visual problems/deficit in children can generally affect their educational performance as well as psychological health, some disorders like Vitamin A deficiency are also common causes of childhood mortality (1,2).

Understanding the causes leading to visual loss in children can guide in many ways to direct resources to prevent the problem. Causes of childhood blindness and visual impairment vary in different regions of the world. Generally, the majority of causes in developing countries are either preventable or treatable. Refractive error is the second (33%) leading cause of low vision in Ethiopia next to cataract (3).

Screening for eye disorders in pre-school and school aged children are routinely done in the developed world. Visual health has been recognized to be inextricably linked to school achievement, quality of life, employability and economic productivity and National School Health and Nutrition Strategy (SHN) has been launched in 2012 by Federal Ministry of Education, Ethiopia (4). Although School Eye Health (SEH) has been included as one of the major focus areas of the strategy, no preschool or school eye screening service has been initiated yet. Ethiopia has achieved over 85% primary school net enrolment ratio of children (5). Therefore, schools provide an excellent opportunity for screening of school age children for ocular disorders in a community. This is important for early detection of eye diseases and prevention of blindness (6).

There are limited studies done on pattern of eye disorders among school age children in Ethiopia. In a population based study done in central Ethiopia, it has been shown that 51.6% of children under 10 years of age suffer from active trachoma (7).

This study was done with the main objective to determine prevalence and pattern of ocular morbidity among school children in Roman Dega Kedida, Southern Nations, Nationalities and Peoples of Ethiopia. There is no previous study conducted in this area.

## Patients and Methods

This cross sectional study was conducted among Roman Dega Kedida primary school children in Kembata Tembaro Zone of Southern Nations, Nationalities and People of Ethiopia (SNNP) in May, 2013. The school is found 350km from Addis Ababa, the capital city of Ethiopia and provides education from grade 1–8. All children who were aged 16 years or less and attending the school during the study period were included.

A structured questionnaire was designed for the purpose of the study. The questionnaire included socio demographic characteristics of children, assessment of visual acuity (VA), and evaluation of children for any ocular problem. Examination of children was done by the primary investigator.

Distant Visual Acuity (VA) was measured with a Snellen illiterate E chart placed at 6 meters in a bright illumination. If the presenting VA was found to be 6/12 or worse in one or both eyes, a pinhole was used to check for improvement. External eye examination, motility and anterior segment examination was done with the use of torch light and magnifying loop while posterior segment examination was done using a direct ophthalmoscope. Diagnosis and classification of trachoma and vitamin A deficiency was according to WHO (8,9). Refractive error was considered when subnormal visual acuity (6/12 or worse) improved with a pinhole test. Data entry and statistical analyses were performed using SPSS software for Windows Version 16 (IBM Corp., New York, NY, USA). A p- value of < 0.05 was considered statistically significant.

The study adhered to the tenets of the Declaration of Helsinki and was conducted after obtaining ethical approval from Department of Ophthalmology; Jimma University. All the clinical data obtained from each child was recorded with coding and no mentioning of identity. Children with active trachoma and bacterial conjunctivitis were given Tetracycline eye ointments and chloramphenicol eye ointments respectively, and prescription was given for other problems. Children with vitamin A deficiency were given Vitamin A capsules while those with trichiasis, refractive errors, cornea opacity and cataract were referred to the nearby eye hospital.

## Results

A total of 778 children participated in this study and underwent eye examination. There were 378 (48.6%) males and 400 (51.4%) females making the female to male ratio of 1.06:1. The mean age was 11.34 years (SD= 2.31). There were 201 (25.8 %%) in the age group 6–9 years, 303 (38.9 %%) children in the age group 10–12 years and 274 (35.2%) aged 13–16 years (Table 1).

**Table 1:** Age and Sex distribution of children in Roman Dega Kedida primary school, 2013: n=778

	SEX		Total
		
AGE (years)	Male	Female	
6–9	96 (12.3%)	105 (13.5%)	201 (25.8%)
10–12	143 (18.4%)	160 (20.6%)	303 (38.9%)
≥13	139 (17.9%)	135 (17.4%)	274 (35.2%)

**Total**	**378 (48.6%)**	**400 (51.4%)**	**778 00.0%)**

A total of 201 (25.8%) children were found to have some form of ocular morbidity. The presence of ocular morbidity was not different between boys 98(25.9%) and girls 103(25.8%) (P =0.96). On the other hand, 55 (27.4%) of those in the age group 6–9 years had ocular morbidity as compared with 100 (33.0%) & 46 (16.8%) in the age groups 10–12 years and 13–16 years respectively. This was statistically significant (P= 0.00). The most common ocular morbidity in this survey was trachoma (10.9%) followed by allergic conjunctivitis (6.3%) and refractive error 37 (4.8%) (Table 2).

**Table 2:** Type of ocular diseases and its distribution by sex in Roman Dega Kedida primary school children, 2013. n=778

Ocular Morbidity	Male frequency (%)	Female frequency (%)	Total
Trachoma	39 (5.0)	46 (5.9)	85 (10.9)
Allergic conjunctivitis	22 (2.8)	27 (3.5)	49(6.3)
Refractive error	19 (2.4)	18 (2.3)	37 (4.8)
Vitamin A deficiency	11 (1.4)	6 (0.8)	17 (2.2)
Bacterial conjunctivitis	2 (0.26)	3 (0.38)	5 (0.6)
Chalazion	2 (0.26)	2 (0.26)	4 (0.5 )
Traumatic corneal opacity	1(0.13)	2 (0.26)	3 (0.39)
Cataract	0 (0.0)	1 (0.13)	1 (0.1 )

**Any one or more of the above**	**98(12.6)**	**103(13.2)**	**201 (25.8%)**

A total of 85 children were found to have trachoma, of whom 49 (56.5%) had active trachoma; trachomatous follicles (TF) and/or trachomatous intense inflammation (TI), while 37 (43.5%) had inactive trachoma; trachomatous scar (TS) or trachomatous trichiasis (TT). The occurrence of trachoma was not different between boys and girls (OR= 0.94, 95%CI 0.42–2.09). On the other hand, 28 (3.6%) of children in the age group 6–9 years had trachoma as compared with 41(5.3%) and 16 (2.1%) of those in the group 10–12 years and 13–15 years respectively. This was statistically significant (P=0.004). Considering active trachoma in the age groups 6–9 years, 15 (7.5%) had TF and 5 (2.5%) had TI.

Allergic conjunctivitis was seen in 49 (6.3%) students; the most common type was seasonal allergic conjunctivitis, which was found in 24 (3.1%) of children followed by VKC13 (1.7%) and perennial allergic conjunctivitis12 (1.5). no statistically significant difference was seen between the age groups (p=0.42). There were 5 cases (0.6%) of bacterial conjunctivitis).

A total of 17 (2.2%) children were found to have xerophthalmia with clinical manifestation of Bitot's spot in 17 (2.2%) and report of night blindness by 3 (0.4%) children (All 3 of the children reporting night blindness had Bitot spot). No statistically significant difference was seen between the age groups (p=0.36) or boys and girls (p=0.18).

According to etiological classification, infectious disorders were the most common causes ocular morbidity, which accounted for 90(44%), followed by non-infection inflammation 49 (24%), and refractive error 37 (18.4%) Table 3).

**Table 3:** Etiological categories of ocular diseases in Roman Dega Kedida primary school, 2013. n=201

Etiological category	Number (%)
Infection	90 (44.8)
Non-infection inflammation	49 (24.4)
Refractive error	37 (18.4)
Nutritional	17 (8.5)
Trauma	4 (1.99)
Others	4 (1.99)

**Total**	**201 (100.0%)**

Visual impairment in one or both eyes was found in 41 (5.3%) children; 9 had unilateral VI and 32 had bilateral VI (Among these 26 (81.3%) with mild VI, 6 (18.8%) moderate to severe VI). The cause of visual impairment was refractive error in 37 (90.2%) children, corneal opacity in 3 (7.3%) children and cataract in 1(2.4%) child. According to the WHO classification, the overall prevalence of mild VI was 3.3% and moderate to severe VI was 0.8%. There was no child with unilateral or bilateral blindness.

## Discussion

A total of 201 (25.8%) school children in our study were found to have ocular abnormality. A similar study done in Butajira town, southern Ethiopia, found 62.6% of school children (5–15 years) to have ocular morbidity (10). Ocular problems were found among 10.2% school children (7–17 years) in rural Tanzania (11), in 15.5% among 4- 24 years old in South-Western Nigeria (12) and in 6.1% among 6–16 years old in rural south-eastern Nigeria(13).

The most common ocular abnormality identified in this study was trachoma 85 (10.9%) followed by allergic conjunctivitis (6.3%) and refractive errors (4.8%). A nation-wide survey among schoolchildren in 2006 in Ethiopia found the overall prevalence of trachoma to be 13%(3). The above study in Southern Ethiopia also found trachoma to be the most prevalent (53.5%) followed by refractive error (11.8%) (10). Trachoma and xerophthalmia were found in (5.56%) and 5.85% children, respectively, in rural Tanzania (11), while Vernal conjunctivitis was the commonest abnormality found in Southern Western and south-eastern Nigeria schools (13). Differences in the types and magnitude of ocular morbidities among different localities can be due to various geographic, socioeconomic factors and show the importance of doing such studies to have baseline information and intervention.

In this study, the overall prevalence of trachoma among the school children was high, and particularly the presence of follicular conjunctivitis in 7.5% of those below 10 years of age is an indicator that the disease is of a public health problem. We have identified a high prevalence of trachomatous scarring (TS) (40%) in this study. A similar high proportion of TS (40%) has also been reported in the school children in Butajira (10). TS represent a sequel of repeated infections and is more prevalent with aging. A similar trend was seen in this study with 24% of children below 9 years having TS as compared with 34% of those above 12 years of age. However, the excess prevalence may represent a subgroup at high risk for the blinding complications of trachoma, as it has been suggested that it may be likely that these children have a different host response to infection (14). This may be supported by the findings of 2 (0.3%) children in our study with trachomatous trichiasis and 14 (0.8%) in the Butajira study (10). The prevalence of trachoma was similar in girls and in boys, similar to the study in Butajira.

Allergic conjunctivitis was seen in 49 (6.3%) students, and VKC was found in 13 (1.7%) of children. A similar prevalence of VKC of 1.6% has been reported in a rural community in Goro (15) while this was 2.9% of children in SE Nigeria (13) and 4.0% in Rwanda had VKC (16).

A total of 17 (2.2%) children were found to have clinical xerophthalmia as demonstrated by Bitot's spot 17 (2.2%) and/ or night blindness, 3 (0.4%). This also classifies the disorder as a public health importance in the school children. Although night blindness is subjective and nonspecific in nature, all children who reported it had associated Bitots spot. The frequency of lesions that are unresponsive to vitamin A and therefore not due to recent vitamin A deficiency increases in school age children. (17,18). Therefore, a limitation of our findings is, apart from the clinical findings, our cases were not supported by biochemical determination of serum vitamin A level. However, the results ask for further studies to explore the underlying causes and further follow-up of patients.

A study of the prevalence of refractive error among school children shows a wide variation in the literature. The lack of standard definitions as well as differences in the age group studied makes comparisons between studies difficult. In our study, we found refractive error to affect 4.8% children and none of the children with refractive error in our study was wearing spectacles. A study among school children in (7–18 years) in Gurage and SiltiZones found a prevalence of 6.3% while a prevalence of 11.8% was reported from Butajira, using a similar definition (10). A study in Gondar town among 4 years to 24 years reported a prevalence of 9% (19).

The main strength of this study is that all of the children attending the school and aged 6–16 years were included. It is not also without limitations. The equipment used for examination was basic that allow diagnosis of the stated disorders. Using better equipment particularly for anterior and posterior segment exam might have revealed some more retinal problems or anterior uveitis that could have been missed. We have not also done subjective and objective refraction for children found to have refractive errors. This prohibits further characterization of the refractive errors. Some eyes with high refractive errors may not improve with pin hole. Despite the limitations, we believe this study complements important evidence on patterns of eye diseases among school children in the region.

Ocular morbidities are prevalent among the school children with trachoma, allergic conjunctivitis and refractive errors being the most common ones. Most of these ocular diseases observed were either preventable or treatable. If some of the disorders were not treated at the right time they may affect the child's performance in the school, or may lead to blindness. Proper health education to the community in general and school staff and student families in particular on the burden of the disorders and ways of prevention and treatment is recommended. Establishing regular school screening program is recommended to make the early diagnosis and treatment of childhood blindness sustainable in the district. A similar study in other schools in the district is also recommended.
